# Predictors of Postoperative Neurological Outcome After Surgical Evacuation of Traumatic Acute Subdural Hematoma: A Retrospective Cohort Study

**DOI:** 10.7759/cureus.117172

**Published:** 2026-09-29

**Authors:** Muhammad Nauman Shahid, Zainab Wasim Sulehri, Mah Noor Shafiq, Hamna Abdul Ghaffar, Mohsin Tahir, Noman Ahmad, Ayesha Khalid, Ali Khalid, Nadia Ishtiaq

**Affiliations:** 1 Department of Anatomy, Lahore Medical and Dental College, Lahore, PAK; 2 Department of Biochemistry, Lahore Medical and Dental College, Lahore, PAK; 3 Department of Medical Sciences, Lahore Medical and Dental College, Lahore, PAK; 4 Department of Neurosurgery, Ghurki Trust Teaching Hospital, Lahore, PAK; 5 Department of Surgery, Ghurki Trust Teaching Hospital, Lahore, PAK; 6 Department of Medicine, Amna Inayat Medical College, Lahore, PAK; 7 Department of Medical Sciences, Al-Aleem Medical College, Lahore, PAK; 8 Department of Medical Sciences, Affiliated Hospital of Hebei University of Engineering, Handan, CHN

**Keywords:** acute subdural hematoma, craniotomy, glasgow coma scale, neurological outcome, prognostic factors, traumatic brain injury

## Abstract

Background: Traumatic acute subdural hematoma (ASDH) is a severe consequence of traumatic brain injury and is associated with substantial mortality and neurological disability among surgically treated patients.

Objective: To identify clinical, physiological, radiological, and operative factors associated with unfavorable three-month neurological outcome and to support risk stratification among patients undergoing surgical evacuation of traumatic ASDH.

Methods: This retrospective cohort study included 120 adults who underwent surgical evacuation of traumatic ASDH at Ghurki Trust Teaching Hospital, Lahore, between March 2025 and March 2026. Demographic, clinical, radiological, and perioperative variables were evaluated. The primary outcome was unfavorable neurological status at three months, defined as a Glasgow Outcome Scale-Extended (GOSE) score of 1-4. Multivariable logistic regression was used to identify factors independently associated with unfavorable outcomes. As all participants underwent surgery and no non-operative comparison group was included, the study was designed to evaluate prognostic associations rather than the effect of surgical evacuation itself.

Results: The mean age was 50.8 ± 17.9 years; 75 (62.5%) patients were male, and 45 (37.5%) were female. An unfavorable neurological outcome occurred in 74 (61.7%) patients, and three-month mortality was 21.7% (26/120). Factors independently associated with unfavorable outcome were admission Glasgow Coma Scale ≤8 (aOR 4.86, 95% CI 2.05-11.50), bilateral non-reactive pupils (aOR 4.10, 95% CI 1.13-14.86), age per 10-year increase (aOR 1.42, 95% CI 1.13-1.79), preoperative hypotension (aOR 2.79, 95% CI 1.01-7.71), midline shift >10 mm (aOR 2.53, 95% CI 1.07-5.98), and compressed or absent basal cisterns (aOR 2.71, 95% CI 1.12-6.57). The model showed apparent good discrimination with an in-sample AUC of 0.86.

Conclusion: Greater neurological injury severity, bilateral nonreactive pupils, preoperative hypotension, increasing age, and CT markers of intracranial mass effect were independently associated with unfavorable three-month neurological outcome among surgically treated patients with traumatic ASDH. These findings may assist postoperative risk stratification but should not be interpreted as evidence of the effectiveness or appropriateness of surgical evacuation.

## Introduction

Traumatic acute subdural hematoma (ASDH) is one of the most severe intracranial consequences of traumatic brain injury (TBI). It is associated with substantial early mortality and long-term neurological disability despite advances in prehospital care, neuroimaging, neurosurgical management, and neurocritical care [1,2]. ASDH commonly results from disruption of bridging veins, cortical vessels, or other intracranial vascular structures during high-energy acceleration-deceleration injury. Neurological deterioration, however, is not determined by hematoma volume alone. Underlying primary brain injury and secondary physiological and intracranial insults, including cerebral swelling, increased intracranial pressure (ICP), impaired cerebral perfusion, hypoxemia, hypotension, and progressive herniation, also influence clinical outcome [2,3]. Consequently, patients with apparently similar hematoma morphology may experience markedly different postoperative neurological courses [4].

Surgical evacuation remains a principal treatment for traumatic ASDH associated with substantial mass effect or neurological deterioration. Current surgical guidance recommends evacuation of an ASDH when hematoma thickness exceeds 10 mm or midline shift exceeds 5 mm on computed tomography (CT), irrespective of the presenting Glasgow Coma Scale (GCS) score; neurological deterioration, pupillary abnormalities, and intracranial hypertension may constitute additional indications in selected patients [5]. Thus, the radiological thresholds themselves are applied independently of GCS, rather than implying that the overall neurological examination is disregarded when determining management. The two principal operative approaches are craniotomy with bone-flap replacement and decompressive craniectomy, in which the bone flap is not immediately replaced. Selection between these procedures depends particularly on the degree of cerebral swelling and intraoperative assessment after hematoma evacuation. The RESCUE-ASDH randomized trial demonstrated broadly similar functional outcomes between the two approaches while showing differences in subsequent cranial procedures and wound-related complications [6]. These findings indicate that operative technique should be interpreted within the context of baseline injury severity, cerebral swelling, and other clinical characteristics rather than as an isolated determinant of neurological outcome [7].

Early prognostication following surgical evacuation of ASDH remains challenging. Mortality and unfavorable functional outcomes have been associated with admission GCS, pupillary reactivity, increasing age, systemic hypotension, hypoxemia, hematoma thickness, midline shift, basal cistern compression, associated cerebral contusions, traumatic subarachnoid hemorrhage, and anticoagulant exposure [6-8]. Several of these variables are also incorporated into established severe TBI prognostic frameworks, including the CRASH and IMPACT models, which integrate neurological examination, age, systemic physiological factors, and CT findings [9,10]. However, models derived from heterogeneous TBI populations may not completely represent patients with ASDH requiring surgical evacuation, in whom acute mass effect, cerebral swelling, herniation, and the requirement for emergency decompression are prominent features of the early clinical course [11].

Previous ASDH studies have frequently emphasized mortality rather than functional neurological recovery, combined surgically and conservatively managed populations, included relatively small cohorts, or evaluated individual predictors without adequately accounting for relationships among clinical and radiological variables [3-6,11]. Interpretation of operative variables is also vulnerable to confounding by indication because patients undergoing decompressive craniectomy or earlier emergency intervention may have greater cerebral swelling or more severe neurological compromise at presentation. Consequently, associations between operative characteristics and adverse outcomes may partly reflect underlying injury severity rather than the independent effect of the procedure itself. Similarly, hematoma thickness, midline shift, and basal cistern compression represent correlated manifestations of intracranial mass effect rather than pathophysiologically independent processes. Their simultaneous evaluation may help determine which measures remain statistically independently associated with neurological outcome after adjustment for other clinical characteristics [12,13].

Identification of patients at increased risk of unfavorable outcome has important clinical implications. Early prognostic information may support communication with patients' families, intensity of postoperative neurocritical monitoring, anticipation of complications, rehabilitation planning, risk adjustment in comparative studies, and, when clinically appropriate, multidisciplinary discussions regarding goals of care and palliative care. Assessment of physiological abnormalities such as hypotension and hypoxemia is also clinically relevant because these variables represent potentially preventable or treatable secondary insults, although an observational prognostic study cannot establish that modifying them will improve outcome [14,15].

Therefore, the present study aimed to identify clinical, physiological, radiological, and perioperative factors associated with three-month neurological outcome among adults who underwent surgical evacuation of traumatic ASDH. The study was designed for prognostic risk stratification within a surgically treated cohort and not to estimate the effectiveness or comparative benefit of surgical evacuation. The primary outcome was functional neurological status assessed using the Glasgow Outcome Scale-Extended (GOSE). We hypothesized that admission neurological severity, pupillary abnormalities, systemic physiological compromise, and radiological markers of intracranial mass effect would remain associated with unfavorable neurological outcome after adjustment for demographic and operative characteristics.

## Materials and methods

Study design and setting

This retrospective cohort study was conducted in the Department of Neurosurgery, Ghurki Trust Teaching Hospital, Lahore, Pakistan, affiliated with Lahore Medical & Dental College and Lahore-UBAS, from March 2025 to March 2026. The study included 120 consecutive adult patients with traumatic acute subdural hematoma (ASDH) who underwent surgical evacuation. Eligible patients were identified retrospectively from institutional neurosurgical and operative records, and all patients fulfilling the predefined eligibility criteria with complete three-month outcome data were included. No selective sampling of individual cases was performed. Ethical approval was obtained from the institutional ethics review committee (ERC/2025/0064B). The study was conducted in accordance with the Declaration of Helsinki and reported according to the Strengthening the Reporting of Observational Studies in Epidemiology (STROBE) Statement [16].

Study population

Patients aged ≥18 years with blunt traumatic brain injury, CT-confirmed acute subdural hematoma, and surgical evacuation by craniotomy or decompressive craniectomy were eligible. Patients with chronic or acute-on-chronic subdural hematoma, spontaneous subdural hemorrhage, penetrating cranial trauma, substantial pre-existing neurological disability, incomplete clinical or radiological records, or unavailable three-month follow-up data were excluded.

Pre-existing severe neurological disability was defined as substantial functional dependence attributable to a neurological disorder before the index traumatic injury. All consecutively identified eligible surgical cases were considered for inclusion. Detailed information regarding smoking, chronic obstructive pulmonary disease, diabetes mellitus, and other microvascular comorbidities was not consistently available in the retrospective dataset and therefore could not be evaluated reliably as prognostic variables. Information regarding subsequent cranioplasty or repeat surgery for recurrent or residual hematoma was also not uniformly captured as a prespecified study variable. The analysis therefore focused on the index surgical evacuation and three-month neurological outcome, and subsequent procedures were not considered separate index cases.

Clinical and radiological assessment

Demographic and clinical variables included age, sex, mechanism of injury, Glasgow Coma Scale (GCS) score on admission, pupillary reactivity, preoperative hypotension, hypoxemia, and antiplatelet or anticoagulant medication use. GCS was assessed according to the scale originally described by Teasdale and Jennett [17], and severe neurological impairment was defined as a GCS score ≤8. The admission GCS score and pupillary findings used for analysis were those documented during the initial neurosurgical assessment. Because of the retrospective design, the available records did not consistently establish whether every neurological examination occurred before intubation, sedative administration, or osmotic therapy. This potential influence on neurological assessment was therefore considered when interpreting these variables.

Preoperative hypotension was defined as documentation of at least one systolic blood pressure measurement <90 mmHg before surgery, whereas hypoxemia was defined as oxygen saturation <90%. The available retrospective records did not permit reliable differentiation between isolated and sustained hypotensive episodes in every patient. Preoperative non-contrast CT was evaluated for maximum hematoma thickness, midline shift, basal cistern status, cerebral contusion, traumatic subarachnoid hemorrhage, intraventricular hemorrhage, and skull fracture. Midline shift was measured at the level of the septum pellucidum and categorized as ≤10 mm or >10 mm. Basal cisterns were categorized as patent or compressed/absent.

Radiological variables were abstracted from the contemporaneous radiological reports and neurosurgical documentation available in the medical records. A standardized blinded re-reading of all CT examinations by a single study assessor was not part of the retrospective protocol; therefore, interobserver agreement could not be evaluated. Hematoma thickness, midline shift, and basal cistern status were considered correlated radiological manifestations of intracranial mass effect rather than pathophysiologically independent processes.

Surgical management

Surgical evacuation was undertaken according to neurological status, radiological mass effect, and clinical deterioration. A hematoma thickness >10 mm or midline shift >5 mm was considered an indication for evacuation irrespective of GCS, while neurological deterioration, pupillary abnormalities, and other evidence of intracranial mass effect were also considered during clinical decision-making. Patients underwent either craniotomy with replacement of the bone flap or decompressive craniectomy when substantial cerebral swelling prevented safe replacement of the bone flap. Procedures were undertaken within the institutional neurosurgical service according to routine departmental practice. Individual surgeon experience and the extent of resident participation were not predefined variables in the retrospective dataset and therefore could not be evaluated as independent predictors of outcome.

The interval from hospital admission to surgery was recorded and categorized as ≤4 hours or >4 hours. This variable represented the hospital admission-to-surgery interval and should not be interpreted as the interval from injury to surgery. Exact injury-to-admission and injury-to-surgery intervals were not consistently available. Likewise, specific reasons for surgery occurring >4 hours after admission, including physiological stabilization, evolving neurological findings, additional diagnostic assessment, operating-room availability, or other logistical factors, were not sufficiently documented to permit reliable subgroup analysis.

Outcome assessment

The primary outcome was neurological status at three months, assessed using the GOSE according to the structured framework described by Wilson et al. [18]. A favorable outcome was defined as GOSE 5-8, whereas GOSE 1-4 was considered unfavorable. This dichotomization was used to distinguish death or severe functional dependence from moderate disability or good recovery and to facilitate comparison with commonly reported functional-outcome analyses in traumatic brain injury. Three-month GOSE outcomes were derived from documented clinical follow-up information. Because of the retrospective study design, a single prospectively designated blinded GOSE assessor and a uniform assessment modality could not be confirmed for every participant; therefore, assessor-related variability could not be excluded.

The GOSE 1-4 category includes clinically distinct outcomes ranging from death to severe disability. The prespecified binary endpoint was retained for the primary analysis. A survivors-only multivariable analysis and full ordinal GOSE regression were not performed because the retrospective dataset did not contain sufficiently standardized patient-level information to reconstruct these analyses reliably. Secondary outcomes included in-hospital mortality, three-month mortality, intensive care unit length of stay, duration of mechanical ventilation, and surgical complications.

Statistical analysis

Data were analyzed using IBM SPSS Statistics for Windows, Version 30 (Released 2024; IBM Corp., Armonk, New York). Continuous variables were summarized as mean ± standard deviation or median with interquartile range, as appropriate, whereas categorical variables were reported as frequencies and percentages. Between-group comparisons were performed using the independent-samples t-test, Mann-Whitney U test, chi-square test, or Fisher's exact test, as appropriate.

Univariable logistic regression was used to evaluate candidate predictors of unfavorable neurological outcome. Candidate variables included age, admission GCS, pupillary reactivity, preoperative hypotension, hypoxemia, hematoma thickness, midline shift, basal cistern status, cerebral contusion, traumatic subarachnoid hemorrhage, antiplatelet or anticoagulant use, operative procedure, and admission-to-surgery interval. Unadjusted odds ratios with 95% confidence intervals were calculated. Variables considered clinically relevant and those with p < 0.10 in univariable analysis were considered for multivariable logistic regression.

The final multivariable model included age, admission GCS, pupillary reactivity, preoperative hypotension, midline shift, and basal cistern status. Adjusted odds ratios with 95% confidence intervals were reported. Hematoma thickness was evaluated together with other radiological measures of mass effect but was not retained in the final adjusted model. Because hematoma thickness, midline shift, and basal cistern status represent correlated manifestations of intracranial mass effect, variables retained after adjustment were interpreted as statistically independent markers within the fitted model rather than as pathophysiologically independent mechanisms.

Multicollinearity was evaluated using variance inflation factors. Model discrimination was assessed using the area under the receiver operating characteristic curve. The final model contained six predictors and 74 unfavorable outcome events, corresponding to approximately 12.3 outcome events per predictor variable. The reported area under the curve represented apparent in-sample discrimination. Bootstrap resampling, cross-validation, and formal optimism correction were not performed; therefore, the model was considered exploratory rather than a validated clinical prognostic tool and requires further internal and external validation. Formal calibration-in-the-large and calibration-slope analyses were not performed. A two-tailed p < 0.05 was considered statistically significant.

Inter-institutional collaboration and author contributions

Although the authors were affiliated with different institutions, this was a single-center study conducted at Ghurki Trust Teaching Hospital, Lahore. Patient identification, clinical management, operative treatment, and source clinical documentation originated from the study center. Data were retrospectively abstracted from institutional medical records by the study team. Collaborating authors contributed to study conception, methodological development, statistical evaluation, interpretation of findings, manuscript preparation, and critical intellectual review.

Formal duplicate independent data extraction and inter-rater agreement assessment were not incorporated into the original study protocol. Statistical analyses were performed using the prespecified SPSS-based analytical approach and were interpreted collaboratively by the authors.

## Results

Study population and baseline characteristics

A total of 120 patients who underwent surgical evacuation of traumatic ASDH were included. The mean age was 50.8 ± 17.9 years; 75 (62.5%) patients were male, and 45 (37.5%) were female. Road traffic collisions were the most frequent mechanism of injury, occurring in 69 (57.5%) patients, followed by falls in 36 (30.0%) and other mechanisms in 15 (12.5%).

Admission GCS ≤ 8 was documented in 67 (55.8%) patients, bilateral nonreactive pupils in 20 (16.7%), preoperative hypotension in 27 (22.5%), and preoperative hypoxemia in 18 (15.0%). Mean hematoma thickness was 15.0 ± 4.7 mm, and mean midline shift was 9.9 ± 4.6 mm. Midline shift >10 mm was present in 49 (40.8%) patients, with compressed or absent basal cisterns in 53 (44.2%), cerebral contusion in 38 (31.7%), and traumatic subarachnoid hemorrhage in 42 (35.0%). Craniotomy was performed in 82 (68.3%) patients and decompressive craniectomy in 38 (31.7%). Surgery was performed within four hours of hospital admission in 89 (74.2%) patients and >4 hours after admission in 31 (25.8%). Baseline characteristics are summarized in Table 1. Complete data for the primary outcome and prespecified core predictors were available for all 120 patients; therefore, no imputation was required.

**Table 1:** Baseline clinical, radiological, and operative characteristics of the study population. Data are presented as mean ± SD or N (%). No inferential statistical test was applied to this descriptive table. GCS: Glasgow Coma Scale; SD: standard deviation.

Characteristic	Total (N = 120)
Age, years	50.8 ± 17.9
Male sex	75 (62.5%)
Female sex	45 (37.5%)
Road traffic collision	69 (57.5%)
Fall	36 (30.0%)
Other mechanism	15 (12.5%)
Admission GCS ≤8	67 (55.8%)
Bilateral non-reactive pupils	20 (16.7%)
Preoperative hypotension	27 (22.5%)
Preoperative hypoxemia	18 (15.0%)
Hematoma thickness, mm	15.0 ± 4.7
Midline shift, mm	9.9 ± 4.6
Midline shift >10 mm	49 (40.8%)
Compressed/absent basal cisterns	53 (44.2%)
Cerebral contusion	38 (31.7%)
Traumatic subarachnoid hemorrhage	42 (35.0%)
Craniotomy	82 (68.3%)
Decompressive craniectomy	38 (31.7%)
Surgery ≤4 hours after admission	89 (74.2%)
Surgery >4 hours after admission	31 (25.8%)

Three-month neurological outcome

At three months, 46 (38.3%) patients achieved a favorable neurological outcome (GOSE 5-8), whereas 74 (61.7%) had an unfavorable outcome (GOSE 1-4). The complete GOSE distribution was death in 26 (21.7%), vegetative state in 4 (3.3%), lower severe disability in 18 (15.0%), upper severe disability in 26 (21.7%), lower moderate disability in 17 (14.2%), upper moderate disability in 13 (10.8%), lower good recovery in 10 (8.3%), and upper good recovery in 6 (5.0%) patients. When grouped into broader functional categories, 30 (25.0%) patients had GOSE 1-2, 44 (36.7%) had GOSE 3-4, 30 (25.0%) had GOSE 5-6, and 16 (13.3%) had GOSE 7-8. Thus, the unfavorable-outcome category comprised both patients who died or remained vegetative and survivors with severe disability and should not be interpreted as a mortality endpoint alone.

Patients with unfavorable outcomes were older than those with favorable outcomes (55.9 ± 17.4 vs. 42.7 ± 16.1 years; p < 0.001). Admission GCS ≤ 8 was present in 55 (74.3%) patients with unfavorable outcomes compared with 12 (26.1%) with favorable outcomes (p < 0.001). Bilateral non-reactive pupils were present in 18 (24.3%) versus 2 (4.3%) patients, respectively (p = 0.004), and preoperative hypotension occurred in 22 (29.7%) versus 5 (10.9%) patients (p = 0.016). Patients with unfavorable outcomes had greater hematoma thickness (16.1 ± 4.8 vs. 13.2 ± 4.0 mm; p < 0.001) and greater midline shift (11.2 ± 4.6 vs. 7.8 ± 3.8 mm; p < 0.001). Midline shift >10 mm occurred in 39 (52.7%) patients with unfavorable outcomes compared with 10 (21.7%) with favorable outcomes (p = 0.001), while compressed or absent basal cisterns were present in 42 (56.8%) and 11 (23.9%), respectively (p < 0.001). Preoperative hypoxemia, cerebral contusion, traumatic subarachnoid hemorrhage, decompressive craniectomy, and surgery >4 hours after admission did not reach conventional statistical significance in the unadjusted group comparisons. Detailed between-group comparisons are presented in Table 2.

**Table 2:** Clinical, radiological, and operative characteristics according to three-month neurological outcome. Data are presented as mean ± SD or N (%). Continuous variables were compared using Welch's independent-samples t-test. Categorical variables were compared using Pearson's χ² test; Fisher's exact test was used when expected cell counts were <5. GOSE: Glasgow Outcome Scale-Extended; GCS: Glasgow Coma Scale; SD: standard deviation.

Variable	Favorable GOSE 5-8 (N = 46)	Unfavorable GOSE 1-4 (N = 74)	p-value
Age, years	42.7 ± 16.1	55.9 ± 17.4	<0.001
Male sex	28 (60.9%)	47 (63.5%)	0.771
Female sex	18 (39.1%)	27 (36.5%)	0.771
Admission GCS ≤8	12 (26.1%)	55 (74.3%)	<0.001
Bilateral non-reactive pupils	2 (4.3%)	18 (24.3%)	0.004
Preoperative hypotension	5 (10.9%)	22 (29.7%)	0.016
Preoperative hypoxemia	4 (8.7%)	14 (18.9%)	0.127
Hematoma thickness, mm	13.2 ± 4.0	16.1 ± 4.8	<0.001
Midline shift, mm	7.8 ± 3.8	11.2 ± 4.6	<0.001
Midline shift >10 mm	10 (21.7%)	39 (52.7%)	0.001
Compressed/absent basal cisterns	11 (23.9%)	42 (56.8%)	<0.001
Cerebral contusion	11 (23.9%)	27 (36.5%)	0.150
Traumatic subarachnoid hemorrhage	12 (26.1%)	30 (40.5%)	0.107
Decompressive craniectomy	10 (21.7%)	28 (37.8%)	0.065
Surgery >4 hours after admission	8 (17.4%)	23 (31.1%)	0.096

Univariable associations with unfavorable neurological outcome

Univariable logistic regression demonstrated strong associations between unfavorable neurological outcome and admission GCS ≤8 (OR 8.20, 95% CI 3.54-18.99; p < 0.001), bilateral non-reactive pupils (OR 7.07, 95% CI 1.56-32.12; p = 0.011), preoperative hypotension (OR 3.47, 95% CI 1.21-9.95; p = 0.021), midline shift >10 mm (OR 4.01, 95% CI 1.74-9.26; p = 0.001), and compressed or absent basal cisterns (OR 4.18, 95% CI 1.84-9.47; p = 0.001). Preoperative hypoxemia, cerebral contusion, traumatic subarachnoid hemorrhage, decompressive craniectomy, and surgery >4 hours after admission showed ORs above unity, although their 95% confidence intervals included 1.0. The available univariable estimates are presented in Table 3.

**Table 3:** Univariable logistic regression for unfavorable three-month neurological outcome. Univariable estimates for categorical predictors were calculated from the reported outcome-stratified frequencies. Continuous-variable univariable ORs for age, hematoma thickness, and continuous midline shift could not be reconstructed reliably from aggregate group summaries alone. Outcome-stratified antiplatelet/anticoagulant exposure data were not available in the analytical results, and therefore, an unverified OR was not reported. OR: odds ratio; CI: confidence interval; GCS: Glasgow Coma Scale.

Candidate predictor	Unadjusted OR	95% CI	p-value
Male sex	1.12	0.52-2.39	0.771
Admission GCS ≤8	8.20	3.54-18.99	<0.001
Bilateral non-reactive pupils	7.07	1.56-32.12	0.011
Preoperative hypotension	3.47	1.21-9.95	0.021
Preoperative hypoxemia	2.45	0.75-7.97	0.136
Midline shift >10 mm	4.01	1.74-9.26	0.001
Compressed/absent basal cisterns	4.18	1.84-9.47	0.001
Cerebral contusion	1.83	0.80-4.18	0.153
Traumatic subarachnoid hemorrhage	1.93	0.86-4.32	0.109
Decompressive craniectomy	2.19	0.94-5.09	0.068
Surgery >4 hours after admission	2.14	0.86-5.31	0.100

Multivariable associations with unfavorable neurological outcome

In multivariable logistic regression, admission GCS ≤8 showed the strongest adjusted association with unfavorable neurological outcome at three months (aOR 4.86, 95% CI 2.05-11.50; p < 0.001). Bilateral nonreactive pupils were associated with approximately fourfold higher adjusted odds of unfavorable outcome (aOR 4.10, 95% CI 1.13-14.86; p = 0.032). Increasing age remained statistically independently associated with unfavorable outcome, with each 10-year increase associated with a 42% increase in adjusted odds (aOR 1.42, 95% CI 1.13-1.79; p = 0.003). Preoperative hypotension was also independently associated with an unfavorable outcome (aOR 2.79, 95% CI 1.01-7.71; p = 0.048).

Among radiological variables, midline shift >10 mm (aOR 2.53, 95% CI 1.07-5.98; p = 0.035) and compressed or absent basal cisterns (aOR 2.71, 95% CI 1.12-6.57; p = 0.027) remained statistically independently associated with unfavorable outcome after adjustment. Hematoma thickness showed an association in the unadjusted comparison but was not retained in the final adjusted model. Because hematoma thickness, midline shift, and basal cistern status represent correlated manifestations of intracranial mass effect, the retained CT variables were interpreted as statistically independent markers within the fitted model rather than pathophysiologically independent processes. The adjusted associations are presented in Table 4.

**Table 4:** Multivariable logistic regression for unfavorable three-month neurological outcome. Binary multivariable logistic regression was performed with an unfavorable three-month outcome (GOSE 1-4) as the dependent variable. The model simultaneously included age, admission GCS ≤8, bilateral non-reactive pupils, preoperative hypotension, midline shift >10 mm, and compressed/absent basal cisterns. CI: confidence interval; GCS: Glasgow Coma Scale; GOSE: Glasgow Outcome Scale-Extended; OR: odds ratio.

Predictor	Adjusted OR	95% CI	p-value
Age, per 10-year increase	1.42	1.13-1.79	0.003
Admission GCS ≤8	4.86	2.05-11.50	<0.001
Bilateral non-reactive pupils	4.10	1.13-14.86	0.032
Preoperative hypotension	2.79	1.01-7.71	0.048
Midline shift >10 mm	2.53	1.07-5.98	0.035
Compressed/absent basal cisterns	2.71	1.12-6.57	0.027

Model performance

The six-predictor multivariable model demonstrated an apparent in-sample area under the receiver operating characteristic curve of 0.86 (95% CI 0.79-0.92), indicating apparent good discrimination within the derivation cohort. With 74 unfavorable outcome events and six retained predictors, the model contained approximately 12.3 events per predictor variable. Variance inflation factors were <2.5, indicating no evidence of important multicollinearity among the retained predictors. The Hosmer-Lemeshow goodness-of-fit test showed no evidence of major lack of fit (p = 0.63). However, bootstrap resampling, cross-validation, and formal optimism correction were not performed; therefore, an optimism-corrected AUC was not available. Calibration-in-the-large and calibration slope were also not estimated. Consequently, the reported AUC represents apparent in-sample discrimination, and the model should be considered exploratory rather than a validated clinical prediction tool.

Secondary outcomes and mortality

In-hospital mortality occurred in 22 (18.3%) patients and increased to 26 (21.7%) by three months. Therefore, 22 of the 26 deaths documented by three months occurred during the index hospitalization, while four additional deaths occurred after hospital discharge and before the three-month assessment. Median intensive care unit stay was seven days (IQR 4-11), and median duration of mechanical ventilation was four days (IQR 2-7). Three-month mortality was higher among patients with admission GCS ≤8 than among those with GCS >8 (22/67 (32.8%) vs. 4/53 (7.5%), respectively; p < 0.001). Detailed cause-specific mortality, exact timing of individual deaths, place of post-discharge death, and information regarding withholding or withdrawal of life-sustaining treatment were not consistently available in the retrospective analytical dataset and therefore could not be reported reliably.

Decompressive craniectomy was more frequent among patients with unfavorable than favorable outcomes (28 (37.8%) vs. 10 (21.7%), respectively), although the unadjusted association did not reach conventional statistical significance (OR 2.19, 95% CI 0.94-5.09; p = 0.068). Similarly, surgery >4 hours after admission occurred in 23 (31.1%) patients with unfavorable outcomes compared with 8 (17.4%) patients with favorable outcomes (OR 2.14, 95% CI 0.86-5.31; p = 0.100). The >4-hour category represented the interval from hospital admission to surgery rather than from traumatic injury to surgery. Continuous admission-to-surgery intervals and the specific reasons for delay were not sufficiently complete for reliable calculation of the mean delayed-surgery interval or cause-specific analysis. Accordingly, operative technique and admission-to-surgery timing were interpreted as not statistically independently associated with neurological outcome in this cohort rather than as evidence that these factors have no clinically important effect. Secondary outcomes and outcome-related comparisons are summarized in Table 5.

**Table 5:** Secondary clinical outcomes and outcome-related comparisons. Data are presented as N (%) or median (IQR), as appropriate. Between-group categorical comparisons were performed using Pearson's χ² test or Fisher's exact test, as appropriate. No inferential statistical test was applied to descriptive overall mortality, ICU stay, or mechanical ventilation duration. GCS: Glasgow Coma Scale; ICU: intensive care unit; IQR: interquartile range.

Outcome/Variable	Value	Comparison	p-value
In-hospital mortality	22 (18.3%)	-	-
Three-month mortality	26 (21.7%)	-	-
ICU stay, days	7 (IQR 4-11)	-	-
Mechanical ventilation, days	4 (IQR 2-7)	-	-
Three-month mortality, GCS ≤8	22 (32.8%)	GCS ≤8 vs. >8	<0.001
Three-month mortality, GCS >8	4 (7.5%)	-	-
Decompressive craniectomy, unfavorable outcome	28 (37.8%)	Unfavorable vs. favorable	0.068
Decompressive craniectomy, favorable outcome	10 (21.7%)	-	-
Surgery >4 hours after admission, unfavorable outcome	23 (31.1%)	Unfavorable vs. favorable	0.100
Surgery >4 hours after admission, favorable outcome	8 (17.4%)	-	-

## Discussion

The present study showed that admission neurological severity, pupillary reactivity, increasing age, preoperative hypotension, and radiological markers of intracranial mass effect were independently associated with unfavorable three-month neurological outcome among patients undergoing surgical evacuation of traumatic ASDH [1]. Admission GCS ≤8 demonstrated the strongest adjusted association, followed by bilateral non-reactive pupils, while midline shift >10 mm and compressed or absent basal cisterns remained statistically significant after adjustment. These findings support the concept that neurological examination, physiological status, and CT-defined mass effect provide complementary prognostic information in surgically treated ASDH [2,3].

Low admission GCS and abnormal pupillary reactivity are well-established indicators of injury severity in traumatic brain injury. Their associations with unfavorable outcomes in the present cohort are consistent with previous ASDH studies and with established prognostic frameworks such as CRASH and IMPACT [9,10]. Pupillary non-reactivity may reflect severe intracranial hypertension, brainstem dysfunction, or evolving herniation, whereas GCS provides a broader assessment of neurological impairment. The persistence of both variables in the adjusted model suggests that they contribute distinct prognostic information, although interpretation should remain cautious because retrospective records could not consistently establish whether all examinations preceded intubation, sedation, or osmotic therapy [4].

Radiological markers of mass effect were also important. Midline shift >10 mm and compressed or absent basal cisterns remained statistically independently associated with unfavorable outcome, whereas hematoma thickness, despite showing an unadjusted association, was not retained in the final multivariable model. This should not be interpreted as demonstrating biological independence among these CT variables because hematoma thickness, midline displacement, and cisternal compression are related manifestations of the same underlying mass-effect process. Rather, midline shift and cisternal status provided additional statistical information after adjustment for the other variables included in the model. These CT findings are also clinically relevant because hematoma thickness and midline shift remain central components of established surgical decision-making in acute SDH [5,6].

Preoperative hypotension was independently associated with an unfavorable neurological outcome. Hypotension can compromise cerebral perfusion in an already injured brain and may aggravate secondary ischemic injury, which is consistent with established principles of severe TBI management [7,8]. Nevertheless, the present study was observational and cannot establish that correction of hypotension would directly improve outcome. Hypoxemia showed an increased unadjusted association but was not retained in the adjusted model; therefore, the results should not be interpreted as demonstrating that all secondary physiological abnormalities independently contributed to the outcome in this cohort [9].

Decompressive craniectomy was more frequent among patients with unfavorable outcomes, but operative technique was not statistically independently associated with outcome after adjustment in this cohort [10]. This observation is compatible with confounding by indication because decompressive craniectomy is often selected in patients with greater cerebral swelling or when replacement of the bone flap is considered unsafe. The RESCUE-ASDH trial similarly emphasized that differences between craniotomy and decompressive craniectomy should be interpreted in relation to functional outcome, subsequent cranial surgery, and procedure-related complications rather than by assuming that one technique is intrinsically responsible for poor neurological recovery [6]. The present study therefore does not establish equivalence between procedures and was not designed to determine which surgical strategy is superior [2].

Surgery performed >4 hours after hospital admission was also more frequent among patients with unfavorable outcomes but was not statistically independently associated with outcome after adjustment. Importantly, the measured interval represented admission-to-surgery time rather than injury-to-surgery time. Injury-to-admission intervals and detailed reasons for delay were not consistently available, making it impossible to distinguish delays related to late presentation, physiological stabilization, evolving neurological deterioration, diagnostic work-up, or operating-room availability. Consequently, the absence of an independent association should not be interpreted as evidence that delay is clinically unimportant, and timely evacuation remains appropriate once a surgical indication has been established [11,12].

The functional outcome findings also require careful interpretation. Although 74 patients were classified as having an unfavorable GOSE of 1-4, this group included 26 deaths, four patients in a vegetative state, and survivors with different degrees of severe disability. Thus, the 61.7% unfavorable-outcome rate is not equivalent to mortality. Three-month mortality was 21.7%, reflecting the substantial severity of injury in this operated cohort and remaining broadly consistent with the substantial mortality reported in surgically managed ASDH populations [1]. However, detailed causes of death, exact timing and location of post-discharge deaths, and decisions regarding withholding or withdrawal of life-sustaining treatment were not consistently available. These missing data limit direct comparison of mortality across cohorts because limitation-of-care practices can materially influence observed mortality [13].

The multivariable analysis should be regarded as an exploratory prognostic assessment rather than as the development of a validated prediction tool. The final model contained six predictors and 74 unfavorable outcome events, corresponding to approximately 12.3 events per predictor, and produced an apparent in-sample AUC of 0.86 [14]. However, no bootstrap resampling, cross-validation, optimism correction, calibration-in-the-large, or calibration-slope analysis was performed. Apparent discrimination may therefore overestimate performance in new patients. Established models such as CRASH and IMPACT underwent broader derivation and validation, emphasizing that independent validation is necessary before a new prognostic model can be applied clinically [9,10].

An important limitation of interpretation is that every patient in this cohort underwent surgical evacuation. There was no conservatively managed or alternative-treatment comparison group; therefore, the study cannot estimate the causal effect of surgery [15]. Neither the observed mortality nor the unfavorable functional-outcome rate should be interpreted as evidence that evacuation was effective or ineffective. Rather, the study describes prognostic associations among patients selected for surgery because of their clinical and radiological presentation. This distinction is particularly important when comparing the present findings with other ASDH cohorts that may differ in referral patterns, injury severity, treatment thresholds, operative selection, and neurocritical-care practices [1,16].

The study has several strengths, including assessment of clinical, physiological, radiological, and operative variables within the same multivariable analysis and use of three-month GOSE rather than mortality alone as the primary functional endpoint [13]. However, the retrospective single-center design introduces potential selection bias, measurement variability, and residual confounding. The dichotomized GOSE endpoint combines death with severe non-fatal disability, and survivors-only and ordinal GOSE analyses were not available. Information regarding smoking, chronic obstructive pulmonary disease, diabetes mellitus, exact injury-to-surgery timing, repeat operations, surgeon-level experience, causes of death, and limitation-of-care decisions was also incomplete [15,16]. Furthermore, three-month follow-up may not capture the full trajectory of neurological recovery after severe TBI. Prospective multicenter studies with standardized 6-12-month assessments, detailed perioperative variables, repeat-procedure reporting, cause-specific mortality, and formal internal and external model validation are therefore warranted [17,18].

## Conclusions

Admission GCS ≤8, bilateral nonreactive pupils, increasing age, preoperative hypotension, midline shift >10 mm, and compressed or absent basal cisterns were independently associated with unfavorable three-month neurological outcomes among patients undergoing surgical evacuation of traumatic ASDH. Midline shift and basal cistern compression should be interpreted as statistically independent markers of intracranial mass effect within the multivariable model rather than as pathophysiologically independent processes. Operative technique and admission-to-surgery interval were not independently associated with outcome in this cohort, but these findings do not exclude clinically important effects of surgical strategy or timing. The identified factors may support early risk stratification, family counseling, postoperative monitoring, and rehabilitation planning. Because the study included only surgically treated patients and the prognostic model was not internally or externally validated, the findings should not be interpreted as evidence of the effectiveness of surgical evacuation and require confirmation in prospective multicenter studies with longer follow-up.
